# Prevalence and incidence of musculoskeletal extremity complaints in children and adolescents. A systematic review

**DOI:** 10.1186/s12891-017-1771-2

**Published:** 2017-10-18

**Authors:** Signe Fuglkjær, Kristina Boe Dissing, Lise Hestbæk

**Affiliations:** 1 Department of Sports Science and Clinical Biomechanics, Faculty of Health Sciences, University of Southern Denmark, Campusvej 55, DK-5230 Odense M, Denmark; 2 Nordic Institute of Chiropractic and Clinical Biomechanics, Campusvej 55, DK-5230 Odense M, Denmark

**Keywords:** Musculoskeletal injury, Musculoskeletal pain, Paediatrics, Prevalence, Incidence

## Abstract

**Background:**

It is difficult to gain an overview of musculoskeletal extremity complaints in childhood although this is essential to develop evidence-based prevention and treatment strategies. The objectives of this systematic review were therefore to describe the prevalence and incidence of musculoskeletal extremity complaints in children and adolescents in both general and clinical populations in relation to age, anatomical site and mode of onset.

**Methods:**

MEDLINE and EMBASE were electronically searched; risk of bias was assessed; and data extraction was individually performed by two authors.

**Results:**

In total, 19 general population studies and three clinical population studies were included with children aged 0-19 years. For most of the analyses, a division between younger children aged 0-12 years, and older children aged 10-19 years was used. Lower extremity complaints were more common than upper extremity complaints regardless of age and type of population, with the most frequent pain site changing from ankle/foot in the youngest to knee in the oldest. There were about twice as many non-traumatic as traumatic complaints in the lower extremities, whereas the opposite relationship was found for the upper extremities in the general population studies. There were relatively more lower extremity complaints in the general population studies than in the clinical population studies. The review showed no pattern of differences in reporting between studies of high and low risk of bias.

**Conclusions:**

This review shows that musculoskeletal complaints are more frequent in the lower extremities than in the upper extremities in childhood, and there are indications of a large amount of non-traumatic low intensity complaints in the population that do not reach threshold for consultation. A meta-analysis, or even a simple overall description of prevalence and incidence of musculoskeletal extremity complaints in children and adolescents was not feasible, due to a large variety in the studies, primarily related to outcome measurements.

**Electronic supplementary material:**

The online version of this article (10.1186/s12891-017-1771-2) contains supplementary material, which is available to authorized users.

## Background

Recently, the Global Burden of Disease studies reported musculoskeletal pain as one of the leading causes of years lived with disability [1] and this constitutes a substantial burden on society [2]. Therefore, it is important to design better prevention strategies and early effective treatment. To do that, more basic knowledge about the epidemiology of musculoskeletal complaints in children and adolescents must be obtained first.

The epidemiology of spinal pain in children is well-described [3–5], whereas less attention has been given to musculoskeletal extremity complaints (MEC) in children. Furthermore, musculoskeletal problems in childhood might not only lead to musculoskeletal complaints in adulthood, but could also be a barrier for physical activity and thus have a negative influence on general health [6]. It has been shown that physical activity is important for health in children and adolescents [6–9], and in addition the amount of physical activity in childhood is considered to be a predictor of the amount of physical activity in adults [9], which is important in prevention of many lifestyle disorders, e.g. diabetes and cardiovascular disease [8, 9].

Various terms have been used in relation to MEC, starting from the less severe ache to injury or more severe musculoskeletal disorders. Commonly, MEC are divided into traumatic and non-traumatic complaints, where a traumatic complaint has been defined as an injury resulting from a specific identifiable event, whereas a non-traumatic complaint is not related to an identifiable event [10]. In research of MEC, focus has traditionally been on either specific groups of athletes, or injuries reported at emergency departments, and patterns of paediatric injuries in relation to sport are therefore well described [11–13]. While this type of research provides valid information about specific injuries, mainly injuries of a traumatic onset, they do not represent the full picture of MEC in the general population. Specifically, none of these methods collect valid information about non-traumatic complaints in the general population, which has been shown to represent a large part, close to two thirds, of MEC [14, 15]. The prevalence and treatment strategies of some types of specific injuries, e.g. fractures [16] and ankle distortions [17–19] are well described, but such knowledge has not been accumulated for many other types of MEC, e.g. overuse injuries and non-specific minor complaints. To inform the development of evidence-based prevention and treatment strategies, the first step is to increase knowledge about types and frequency of MEC in childhood. At present it is difficult to gain an overview of the extent of various types of complaints in relation to mode of onset (traumatic vs. non-traumatic), different anatomical locations and histological involvement. Therefore, the objectives of this systematic review were to investigate the prevalence and incidence of musculoskeletal extremity complaints in children and adolescents in both general and clinical populations in relation to age, distribution between different complaint sites, and types of complaints (traumatic vs. non-traumatic). Furthermore, differences between general and clinical populations will be explored.

## Methods

### Case terminology

Different traditions and interest in different levels of complaint-severity are reasons for various terms used in research about musculoskeletal extremity complaints: injury, disorder, discomfort, complaint, pain, ache etc. In this review we analysed musculoskeletal extremity complaints in the general population, and therefore found a broad term of musculoskeletal extremity complaint (MEC) to be most comprehensive and this will be used throughout the rest of this article, regardless of the term used in the referenced articles. When possible, the MEC were divided according to causation, and were categorised as either traumatic or non-traumatic. A traumatic complaint was defined as an injury resulting from a specific identifiable event, whereas a non-traumatic episode was not related to an identifiable event [10]. For example, a traumatic complaint could be pain due to fall from a horse, and a non-traumatic complaint could be pain of unspecific origin developed over a longer period of time.

### Identification of studies

The search was made in collaboration with a research librarian. Two electronic databases, MEDLINE and EMBASE, were searched for articles published before September 2015. The following search terms were used both as MeSH terms and free text in MEDLINE, and as Subject heading and abstract term in EMBASE: “prevalence”, “incidence”, “musculoskeletal disorder”, “musculoskeletal injury”, “musculoskeletal pain”, “extremity”, “limb”, “children”, “adolescents”, “paediatric”. As free text/abstract term “toddlers” and “teenager” were searched as well. Different forms of spelling and synonyms were used. The full search strategy is seen in Additional file 1. In addition, the reference lists of the relevant obtained articles were screened for additional relevant articles.

### Inclusion criteria

The article had to report the prevalence or incidence of musculoskeletal disorders, complaints, injuries, pain or other description of complaints in the upper and/or lower extremities in general or clinical populations of children and adolescents. All levels of prevalence and incidence rates were included, and could be both parental reported or self-reported values. All study designs were included, but they had to be published in English, Swedish, Norwegian or Danish.

### Exclusion criteria

Special settings or groups, e.g. children with other diseases such as diabetes or other chronic diseases, or children from a specific sport setting (e.g. football players) were excluded, because their pattern of injuries might not be comparable to rest of the population.

### Selection of studies

The first and third author (SF and LH) reviewed the titles and abstracts and identified relevant articles to be read in full text. Inclusion of articles based on full text was decided by agreement between the first and third author (SF and LH).

### Assessment of quality

We were not aware of quality assessment tools specifically designed for studies of prevalence and/or incidence, since most quality assessment tools are designed for studies of associations or comparative effectiveness in either observational studies [20–23] or randomised clinical trials [24]. Therefore the quality of the included articles was assessed by a modified version of the Quality In Prognosis Studies (QUIPS) tool (Additional file 2), which was originally developed to assess bias in studies of prognostic factors [25]. This tool identifies six domains to consider when evaluating risk of bias in studies of prognostic factors: ‘Study Participation’, ‘Study Attrition’, ‘Prognostic Factor Measurement’, ‘Outcome Measurement’, ‘Study Confounding’ and ‘Statistically Analysis and Reporting’ [25]. Three of these domains: ‘Study Participation’, ‘Outcome Measurement’ and ‘Statistical Analysis and Reporting’ were considered to be important, relevant and adequate for studies investigating prevalence, whereas the attrition domain was also included for studies investigating incidence, due to the main interest in relation to representativeness of the population of interest and the validity of the outcome measurement. In the ‘Statistical Analysis and Reporting’ domain, the items “strategy for model building is appropriate and is based on a conceptual framework or model” and “the selected statistical model is adequate for the design of the study” were not relevant and therefore ignored in the assessment. ‘Study Attrition’ is irrelevant for cross-sectional studies of prevalence and was therefore not included in the assessment of these studies, and ‘Prognostic Factors Measurement’ and ‘Study Confounding’ were not relevant either, since our results did not include analyses of prognosis or associations. The domains are described in detail by Hayden et al. [25].

Each of the three (four) domains was categorized as “yes”, “partly” or “no” according to whether the level of quality was adequately fullfilled or not. In evaluation of risk of bias in studies of prevalence and incidence, the most important parameter to consider is whether the study sample is representative of the source population, thus leaving ‘Study Participation’ and ‘Study Attrition‘ the most important domains in this review. This is supported by ‘Study Participation’ being the most frequently used parameter across different tools of evaluation of quality and susceptibility to bias in observational studies [26]. Therefore, ‘Study Participation’ and ‘Study Attrition‘ (if included) needed to be considered as satisfactory (“yes”) for the study to be classified in the low risk of bias category. Furthermore, the other two domains had to be at least partly fulfilled. If ‘Study Participation’ and ‘Study Attrition’ (if included) was not considered satisfactory (“no”) the study was categorized as high risk of bias, regardless the judgements in the other domains. If ‘Study Participation’ and ‘Study Attrition’ (if included) was only partly fulfilled and both the other domains were not fulfilled, the study was classified as high risk as well. All other combinations were considered to be medium risk of bias.

### Data extraction

The studies were divided into general population studies and clinical population studies. Data were extracted to a descriptive table, a modified version of the STROBE statement [27]. The following relevant items from the STROBE Statement were included in the table: study design, setting, age, study size including response rates, data sources/types of measurement, area of complaint and main results. In addition, type of prevalence or incidence, mode of data acquisition and the level of bias were added to the table.

### Review process

Data extraction and assessment of quality were individually performed by two authors (SF and KBD) and results compared. In case of disagreement, the third author (LH) was consulted and consensus reached. The review was conducted according to the PRISMA guidelines, and the PRISMA checklist can be seen in Additional file 3.

## Analyses

### General population and clinical population studies

Obviously, clinical populations cannot be used to assess prevalence of disease. However, they can be used to identify the type of complaints, for which care is sought. Therefore both general population studies and clinical population studies were included in the review, but data were reported separately.

### Follow-up studies considered as two cross-sectional studies

Some of the general population studies had a longitudinal design. Due to the focus on prevalence and incidence rates, these studies were considered as series of cross-sectional studies and each time point reported separately. If the study included any type of intervention, only baseline estimates were reported.

### Data evaluated in relation to age

It is known that the prevalence of MEC change with age [28]. Therefore, to give the most relevant description, results were reported by age groups. Cut points between age groups were based on reporting in the included articles.

### Prevalence and incidence of MEC reported by anatomical site

In *the general population studies* the use of self-reported questionnaires by children and parents did not make it possible to report specific diagnoses, and therefore the results in this review were simply reported by anatomic site. Wrist, hand and fingers were combined into one anatomic site called “wrist/hand/fingers”, and likewise for the ankle and foot, called “ankle/ft”.

To make the reported findings comparable to the general population studies, the diagnoses found in *the clinical population studies* were converted into similar anatomical sites.

### Distribution of non-traumatic versus traumatic complaints

Where possible, the difference between rates of non-traumatic and traumatic complaints was reported.

### Distribution of lower extremity complaints versus upper extremity complaints

Some of the studies reported combined prevalence or incidence rates of the complete upper or lower extremity regions. To identify a possible pattern in distribution of MEC, ratios between the prevalence or incidence rates of the upper and lower extremities were calculated.

## Results

**Fig. 1:**
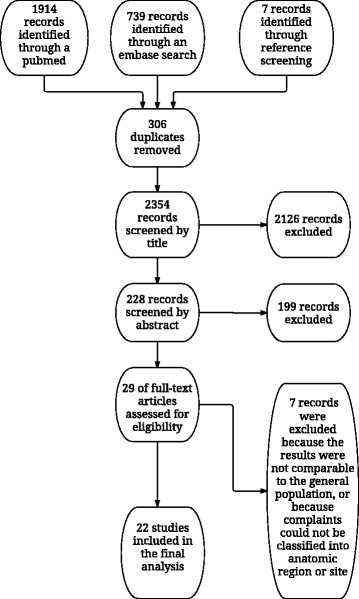
Prisma flow chart outlining the literature search and study selection

In total, 2660 titles were found in MEDLINE, EMBASE and by reference searches. After checking for duplicates and screening of titles and abstracts, 29 articles were found assessable for full-text review. Seven of these were subsequently excluded because results were not representative of the general population [16, 29–32], or because classification into anatomic region or site was not possible [33, 34], thus 22 studies were included in the final analyses (Fig. 1).

### Assessment of quality

The quality assessment categorized fourteen articles to have low risk of bias [14, 15, 35–46], two to have medium risk of bias [47, 48] and six as having high risk of bias [49–54] (Table 1). The clinical population studies were all in the low risk category. There was no disagreement in the independent quality assessments by the two authors (SF and KBD) regarding most of the articles, with the exception of two instances [47, 50] within the ‘study participation’ domain which were solved through discussion without need of mediation by the third author.

**Table 1:** Results of the quality assessment of the 22 included articles

Author and year of publication	Study Participation	Study Attrition	Outcome Measurement	Statistical Analysis and Reporting	Risk of bias assessment
General population studies					
Abujam et al. 2014 [49]	No	N/A	Yes	Yes	High
Adams et al. 2013 [35]	Yes	N/A	Yes	Yes	Low
Auvinen et al. 2009 [50]	No	N/A	Partly	Yes	High
Bishop et al. 2012 [51]	No	N/A	Yes	Yes	High
Diepenmaat et al. 2006 [36]	Yes	N/A	Yes	Yes	Low
Ehrmann Feldman et al. 2002 [37]	Yes	Yes	Partly	Yes	Low
El-Metwally et al. 2006 [15]	Yes	N/A	Yes	Yes	Low
Hoftun et al. 2011 [38]	Yes	N/A	Yes	Yes	Low
Hulsegge et al. 2011 [47]	Partly	Yes	Partly	Yes	Medium
Jespersen et al. 2014 [14]	Yes	Yes	Yes	Yes	Low
Jespersen et al. 2015 [39]	Yes	N/A	Yes	Yes	Low
Krul et al. 2009 [40]	Yes	N/A	Yes	Yes	Low
Mikkelsson et al. 1997 [41]	Yes	N/A	Yes	Yes	Low
Molgaard et al. 2011 [48]	Partly	N/A	Yes	Yes	Medium
Rathleff et al. 2013 [42]	Yes	N/A	Yes	Yes	Low
Shrier et al. 2001 [43]	Yes	Yes	Partly	Yes	Low
Slowinska et al. 2015 [68]	No	N/A	Yes	Partly	High
Smedbraten et al. 1998 [52]	No	N/A	Yes	Yes	High
Verhagen et al. 2009 [54]	Yes	No	Yes	Yes	High
Clinical studies					
Bot et al. 2005 [44]	Yes	N/A^a^	Yes	Partly	Low
Henschke et al. 2014 [45]	Yes	N/A^a^	Yes	Yes	Low
Van der Waal et al. 2006 [46]	Yes	N/A^a^	Yes	Yes	Low

^a^Attrition bias may be present, but very small. If present, it will be on general practitioners level, and not on patient level

### Description of included articles

The search resulted in 19 general population studies and three clinical population studies (Table 2). All the included studies covered children and adolescents of both sexes. Most of the studies were conducted in the northern part of Europe, but also other parts of the world were represented with three studies from North America [35, 43, 55], one from Australia [45] and one from India [49]. Of the 19 general population studies, seven were prospective, four with one follow up evaluation [37, 43, 50, 51], and three studies reporting incidence over time [14, 39, 54]. In most of the studies, data were collected via questionnaires, either self-reported or filled in by parents, but telephone interviews, mobile phone text messages and diagnoses from general practice were also used. The 22 studies used 14 different outcome measurements such as point prevalence, twelve month prevalence and incidence per 1000 exposure hours. Estimates are simply presented in the tables as they appear in the original articles because prevalence and incidence rates were reported in very different ways. For the same reason, meta-analysis was not possible, and even a reliable range of an estimate of frequency, i.e. incidence or prevalence, could not be presented either. However, the findings relating to *differences* in frequency between different age groups, between different anatomical sites and between different populations, are independent of the absolute prevalence or incidence rates and are therefore presented in detail. We attempted to report findings of younger children and older children separately, but due to different cut points in age between the studies, it was not possible to do this. Throughout most of the analyses, a division between younger children aged 0–12, and older children aged 10–19 was used, but to accommodate all studies, some results were reported for the following age groups: 0–9, 9–12, 10–19, 6–17 and 2–19. A summary of findings is presented in Tables 3, 4, 5 and 6.

**Table 2:** Summary of study characteristics of the 22 included studies

Author and year of publication	Study Design	Setting	Age/ grade	Measurements (how did they ask?)	Study Size	Baseline response rate	Follow up response rate	Prevalence/incidence	Type of complaints	Main result (text)	Bias
General population studies											
Abujam et al. 2014	Cross-sectional	India School based	6–17 yrs	Questionnaire filled in by children or parents if the child was less than 14 years	2059	?		Lifetime prevalence	Heel/ Ankle pain or swelling	N (%) 72 (3.5%)	High
Adams et al. 2013	Cross-sectional	US Cohort - Kaiser Permanente Southern California	2–19 yrs	ICD-9 codes	913,178			1 yrs. prevalence	Lower extremity Injuries or pain Upper extremity: Injuries or pain	5.8% 5.1%	Low
Auvinen et al. 2009	Prospective cohort	Finland 2-years Birth Cohort	16–18 yrs	Self-reported questionnaire	7344	7344/ 9215 (80%)	2012/ 2969 (68%)	6-month prevalence	Shoulder pain Elbow pain Wrist pain Knee pain Ankle pain	Prevalence % (N) F: female, M: male Age: 16 yrs. 18 yrs. F: 52 (506) 63 (616) M: 33 (253) 40 (307) F: 2 (22) 4 (35) M: 5 (37) 5 (37) F: 16 (156) 23 (219) M: 15 (114) 20 (150) F: 18 (178) 21 (201) M: 18 (141) 17 (129) F: 16 (150) 19 (179) M: 18 (142) 15 (116)	High
Bishop et al. 2012	Prospective cohort	England Cohort -Avon Longitudinal Study of Parents and Children	5–13 yrs	Questionnaire filled in by chief carer	9380	9380/ 13,988 (67%)	6502/ 13,988 (46%)	Often prevalence	Total replied, n(%) arms, n(%) leg(s), n(%) Total replied, n(%) arms, n(%) leg(s), n(%) Total replied, n(%) arms, n(%) leg(s), n(%)	Age 5* Age 6* 9380 (100.0) 8599 (100.0) 13 + 227 = 240 (2.6) 14 + 299 = 313 (3.6) 1142 + 227 = 1369 (14.6) 1204 + 299 = 1503 (17.5) Age 7* Age 8* 8325 (100.0) 7872 (100.0) 10 + 333 = 343 (4.1) 13 + 475 = 488 (6.2) 1226 + 333 = 1559 (18.7) 1188 + 475 = 1663 (21.1) Age 11* Age 13*6996 (100.0) 6502 (100,0) 22 + 635 = 657 (9.4) 33 + 730 = 763 (11.7) 1386 + 635 = 2021 (28.9) 1342 + 730 = 2072 (31.9)	High
Diepenmaat et al. 2006	Cross-sectional	Holland School based questionnaire	12–16 yrs	Self-reported questionnaire	3485	3485/ 4898 (71%)		Monthly prevalence, pain lasting a day or longer	Arm pain	Male: 4.2% Female: 3.6%	Low
Ehrmann Feldman et al. 2002	Prospective cohort	Canada High school based	7.-9. grade	Self-reported questionnaire	810	810/ 948 (85%)	502/ 810 (62%)	6 months and 12 months Incidence	Upper limb Arm Shoulder	6 months^b^ 12 months^b^ 19.9% 13.3% 9% 7% 8% 5%	Low
El-Metwally et al. 2006	Cross-sectional	Finland 2-years Birth Cohort	9–11 yrs	Self-reported questionnaire	1756	1756/ 1823 (96%)		Weekly prevalence, within the last 3 months	Prevalence N(%) Lower limb pain Ankle-foot pain Knee pain Thigh pain Leg pain Hip pain	Traumatic Non-traumatic Both groups 105 (6.0) 216 (12.3) 321 (18.3) 32 (1.8) 154 (8.8) 186 (10.6) 37 (2.1) 181 (10.3) 218 (12.4) 15 (0.9) 166 (9.5) 181 (10.3) 30 (1.7) 89 (5.1) 119 (6.8) 13 (0.7) 47 (2.7) 60 (3.4)	Low
Hoftun et al. 2011	Cross-sectional	Norway Health Study	13–18 yrs	Self-reported questionnaire	7373	7373/ 7913 (93%)		Weekly prevalence, within the last 3 months	Upper extremity Lower extremity	13–15 yrs. (n % 95% CI): 16–18 yrs. (n % 95% CI): 163 4.0 (3.4–4.6) 129 3.9 (3.3–4.6) 522 12.8 (11.8–13.8) 282 8.6 (7.6–9.6)	Low
Hulsegge et al. 2011	Cross-sectional	Holland Cohort of pregnant women - Prevention and Incidence of Asthma and Mite Allergy	11 yrs	Questionnaire answered by parents and child	2638	2638/ 3963 = (67%)		1-year prevalence	Upper extremity Lower extremity	4,8% (Boys: 3.1%, girls: 6.3%) 10,9% (Boys: 10.4%, Girls: 11.2%)	Medium
J espersen et al. 2014	Prospective	Denmark School based cohort - Childhood Health, Activity and Motor Performance School Study	6–12 yrs	Repeated text messages and ICD-10 diagnosis given by clinicians	1259	90% for sports schools 71% for normal schools		Weekly mean incidence Weekly mean prevalence	Upper extremity Lower extremity Upper extremity Lower extremity	Injury: 0.2 (± 4.0) Overuse injury: 0.04 (± 2.0) Traumatic injury: 0.1 (± 3.5) Injury: 1.0 (± 9.7) Overuse injury: 0.7 (± 8.2) Traumatic injury: 0.3 (± 5.2) Injury: 0.5 (± 7.2) Overuse injury: 0.2 (± 4.9) Traumatic injury: 0.3 (± 5.8) Injury: 4.1 (± 19.9) Overuse injury: 3.2 (± 17.7) Traumatic injury: 1.1 (± 10.4)	Low
Jespersen et al. 2015	Prospective	Denmark School based cohort - Childhood Health, Activity and Motor Performance School Study	6–12 yrs	Repeated text messages and ICD-10 diagnosis given by clinicians	1259	90% for sportsschools 71% for normal schools		Incidence Rate	Shoulder/ Upper Arm Elbow/under Arm Hand/wrist Finger Hip/Groin Thigh Knee Lower leg Achilles Heel Ankle Foot	Overuse Traumatic0.03 (0.02–0.05) 0.03 (0.02–0.04) 0.01 (0.00–0.02) 0.02 (0.01–0.03)0.01 (0.00–0.01) 0.08 (0.06–0.10)0.00 (0.00–0.01) 0.05 (0.03–0.07)0.05 (0.04–0.07) 0.01 (0.00–0.01)0.04 (0.03–0.06) 0.03 (0.02–0.04)0.31 (0.27–0.35) 0.09 (0.07–0.11)0.05 (0.03–0.06) 0.01 (0.00–0.02) 0.06 (0.04–0.08) 0.000.36 (0.31–0.4) 0.000.01 (0.00–0.01) 0.18 (0.15–0.21)0.09 (0.07–0.11) 0.07 (0.05–0.09)	Low
Krul et al. 2009	Cross-sectional	Holland -Second Dutch National Survey in generel practice	2–17 yrs	Interview aboutself-reported musculoskeletal symptoms. In children younger than 12 years interview was carried out with a parent.	2459	2459/ 2719 (90%)		2 weeks prevalence	Upper extremity All ages 2–11 y 12–17 y Lower extremity All ages 2–11 y 12–17 y Hip and knee All ages 2–11 y 12–17 y Ankle and foot All ages 2–11 y 12–17 y	% (n) 1.2 (26) 0.4 (5) 2.7 (21) 6.9 (147) 4.1 (57) 12.4 (90) 3.5 (75) 1.8 (25) 6.5 (50) 3.4 (72) 2.4 (32) 5.5 (40)	Low
Mikkelsson^a^ et al. 1997	Cross-sectional	Finland -School based cohort	3.-5. grade	Self-reported questionnaire	1756	1756/ 2141 (82%)	1628/ 1756 (92%)	Weekly prevalence Relative frequency (%) with 95% CI	Upper extremity Lower extremity	Boys: 7 (6–9)^b^ Girls: 5.5 (4–7)^b^ Boys: 19 (16–22)^b^ Girls: 18 (15–20)^b^	Low
Molgaard et al. 2011	Single-blind case control (case-cohort)	Denmark High School based	16–18 yrs	Self-reported questionnaire	299	227/ 299 (76%)		Monthly-prevalence	Non-traumatic knee pain	57/227 = (25%)	Medium
Rathleff et al. 2013	Cross-sectional	Denmark – Cohort - Adolescent Pain in Aalborg 2011,	12–19 yrs	Self-reported questionnaire	4007	2953/ 4007 (73%)		Point prevalence	Knee Shoulder Foot Shin Hip/groin Forearm/hand Thigh Elbow	Any frequency: 32.3 (30.6–34.0) 13.3 (12.1–14.5) 11.5 (10.4–12.7) 6.2 (5.4–7.1) 5.9 (5.0–6.7) 4.4 (3.7–5.1) 2.7 (2.2–3.2) 2.6 (2.1–3.2)	Low
Shrier et al. 2001	Prospective cohort	Canada High School based	12–18 yrs	Self-reported questionnaire	810	810/948 (85%)	502/810 (62%)	Incidence 6 months 12 months (once a week the last 6 months)	Lower extremity Foot/ankle Knee Leg Hip	6 months^b^ 12 months^b^ % % 21 16 14 8 13 11 11 6 7 4	Low
Smedbraten et al. 1998	Cross-sectional	Norway School based	10–15 yrs	Self-reported questionnaire	569	569/661 (86%)		Prevalence Usually pain	Shoulder Elbow Hand Hip Knee Ankle Foot	Boys (*n* = 282) Girls (*n* = 287) % % 6 14 4 4 5 7 5 7 32 29 7 12 8 11	High
Slowinska et al. 2015	Cross-sectional	Poland School based	6–7 yrs	Questionnaire filled in by parents	1509	1509/ 2748 (55%)		Prevalence often	Knee Hands	Often 11% 3%	High
Verhagen et al. 2009	Prospective cohort	Holland school based (iplay study)	10–12 yrs	Injuries during physical activity monitored by teachers	996/ (996 + 95) (91%)			Incidence Rate	Shoulder Upper arm/elbow Lower arm/wrist/hand Upper leg/hip Knee Lower leg/ankle Foot	0.03 × 0.48 = 0.01^c^ 0.03 × 0.48 = 0.01^c^ 0.22 × 0.48 = 0.11^c^ 0.06 × 0.48 = 0.03^c^ 0.11 × 0.48 = 0.05^c^ 0.25 × 0.48 = 0.12^c^ 0.15 × 0.48 = 0.07^c^	High
Clinical population studies											
Bot et al. 2005	Cross-sectional	Holland Second Dutch National Servey of generel practice	0–19 yrs	ICPC codes	375,899(all ages)			Incidence per 1000 person years	L08 Shoulder complaint L09 Arm complaint L10 Elbow complaint L11 wrist complaint L12 Hand/finger complaint L92 Shoulder syndrome	Age group (years) Sex 0–9 (95% CI) 10–19 (95% CI) M: 0.6 (0.3–0.9) M: 4.0 (3.2–4.9) F: 0.7 (0.4–1.1) F: 4.8 (3.9–5.7) M: 2.0 (1.4–2.6) M:2.3 (1.7–3.0) F:2.3 (1.7–2.9) F: 2.8 (2.1–3.5) M: 1.3 (0.9–1.8) M: 2.3 (1.7–3.0) F: 1.7 (1.2–2.3) F: 1.9 (1.3–2.4) M: 1.8 (1.3–2.4) M: 6.3 (5.3–7.4) F: 2.1 (1.5–2.7) F: 7.9 (6.8–9.1) M: 5.0 (4.1–5.9) M: 8.7 (7.5–9.8) F: 3.9 (3.1–4.7) F: 9.1 (7.8–10.3) M: 0.1 (0.0–0.3) M: 1.4 (0.9–1.9) F: 0.0 ((0.0–0.1) F: 1.9 (1.3–2.4)	Low
Henschke et al. 2014	Cross-sectional	Australia national study in general practice – Bettering the Evaluation and Care of Health	5–17 yrs	ICPC-2 codes	65,279 encounters			Management rate per 100 encounters (encs.)	Lower Limb Upper Limb Lower Limb Upper Limb Lower Limb Upper Limb	Age: 5–9 Boys Girls Number 118121 Rate/100 encs. 1.72 1.85 (95% CI) (1.39–2.06) (1.51–2.19) Number 89 90 Rate/100 encs. 1.30 1.38 (95% CI) (1.02–1.58) (1.09–1.66) Age: 10–14 Boys Girls Number 311254 Rate/100 encs 5.33 4.40 (95% CI) (4.72–5.95) (3.85–4.96) Number 257188 Rate/100 encs 4.41 3.26 (95% CI) (3.85–4.97) (2.76–3.76) Age: 15–17 Boys Girls Number 169134 Rate/100 encs 4.55 2.26 (95% CI) (3.84–5.26) (1.87–2.65) Number 169 82 Rate/100 encs. 4.55 1.38 (95% CI) (3.83–5.27) (1.08–1.69)	Low
van der Waal et al. 2006	Cross-sectional	Holland Second Dutch National Servey of generel practice	0–19 yrs	ICPC codes	375,899(all ages)			Incidence per 1000 person years	L13 Hip complaints L14 Leg/thigh complaints L15 Knee complaints L16 Ankle complaints L17 Foot/toe complaints L77 sprain of ankle/ft L78 sprain/strain of knees Acute meniscus/ligament knee L97 Chronic int knee derangement	Age group (years) 0–9 (95% CI) 10–19 (95% CI) M: 4.0 (3.2–4.8) M: 2.1 (1.5–2.7) F: 4.9 (3.9–5.8) F: 2.2 (1.6–2.8) M: 4.1 (3.3–4.9) M: 5.1 (4.2–6.0) F: 4.2 (3.4–5.1) F: 4.3 (3.4–5.1) M: 3.6 (2.8–4.3) M: 17.0 (15.3–18.6) F: 2.7 (2.1–3-4) F: 16.7 (15.0–18.3) M: 2.4 (1.8–3.0) M: 5.3 (4.4–6.2) F: 1.9 (1.3–2.5) F: 5.0 (4.1–5.9) M: 9.2 (8.0–10.4) M: 16.2 (14.6–17.8) F: 8.5 (7.2–9.7) F: 14.3 (12.7–15.8) M: 3.6 (2.8–4.3) M: 15.4 (13.8–17.0) F: 3.8 (3.0–4.6) F: 16.4 (14.7–18.0) M: 0.9 (0.54–1.33) F: 6.0 (5.0–7.0) F: 0.4 (0.1–0.7) F: 3.7 (2.9–4.5) M: 0.1 (−0.03–0.2) F: 1.2 (0.8–1.7) F: 0 F: 0.8 (0.5–1.2) M: 0.4 (0.1–0.6) F: 4.3 (3.4–5.1) F: 0.6 (0.3–0.9) F: 7.0 (5.9–8.1)	Low

ICD-10: International Classification of Diseases – 10

ICPC: International Classification of Primary Care

^a^ Only baseline were reported

^b^ percentages taken from Fig. 1 in the original article

^c^ percentage taken from Fig. 1 in the original article times the total incidence rate per 1000 exposure hours (0.48)

**Table 3:** General population studies. Prevalence and incidence rates of musculoskeletal extremity complaints in the younger age groups

	0–9 years of age					9–12 years of age			2–19 years of age
	Incidence		Prevalence, %			Incidence	Prevalence, %		Prevalence, %
Anatomic Region/ Site	Weekly, %	IR^a^	Often	Weekly	12 months	IR^a^	2 weeks	Weekly	12 months
Upper extremity in general	0.2 [14]		**2.6–6.2** [51]	0.5 [14]	4.8 [47]		0.4 [40]	5.5–7 [41]	5.1 [35]
Shoulder		0.06 [39]				0.01 [54]			
Elbow		0.03 [39]				0.01 [54]			
Wrist/hand/fingers		0.14 [39]	**3** [53]			0.11 [54]^b^			
Lower extremity in general	1.0 [14]		**14.6–21.1** [51]	4.1 [14]	10.9 [47]		4.1 [40]	18.3 [15]18–19 [41]	5.8 [35]
Hip/groin		0.06 [39]				0.03 [54]		3.4 [15]	
Thigh		0.07 [39]						10.3 [15]	
Knee		0.40 [39]	**11** [53]			0.05 [54]	1.8 [40]^c^	12.4 [15]	
Shin		0.12 [39]							
Ankle/ft		0.71 [39]				0.19 [54]^d^	2.4 [40]	10.6 [15]	

[x]: reference number of included study

Bold: the study received high risk of bias in the quality assessment

^a^ Incidence Rate per 1000 physical activity units

^b^ Anatomical site relates to both lower arm, wrist, hand and fingers

^c^ Anatomical site relates to both hip and knee

^d^ Anatomical site relates to both shin and ankle/ft

**Table 4:** General population studies. Prevalence and incidence rates of musculoskeletal extremity complaints in the older age groups

	10–19 years of age								6–17 years of age
	Incidence, %		Prevalence, %						Prevalence, %
Anatomic Region/Site	6 months	12 months	Point	Often/ usually	Weekly	2 weeks	Monthly	6 months	Lifetime
Upper extremity in general	19.9 [37]	13.3 [37]		**9.4–11.7** [51]	3.9–4.0 [38]	2.7 [40]	3.6–4.2 [36]		
Shoulder	8 [37]	5 [37]	13.3 [42]	**6–14** [52]				**33–63** [50]	
Arm	9 [37]	7 [37]							
Elbow			2.6 [42]	**4** [52]				**2–5** [50]	
Wrist/hand/fingers			4.4 [42]	**5–7** [52]				**15–23** [50]	
Lower extremity in general	21 [43]	16 [43]		**28.9–31.9** [51]	8.6–12.8 [38]	12.4 [40]			
Hip/groin	7 [43]	4 [43]	5.9 [42]	**5–7** [52]					
Thigh			2.7 [42]						
Knee	13 [43]	11 [43]	32.3 [42]	**29–32** [52]		6.5 [40]^a^	25 [48]	**17–21** [50]	
Shin			6.2 [42]						
Ankle/ft	14 [43]	8 [43]	11.5 [42]	**7–12** [52]		5.5 [40]		**15–19** [50]	**3.5** [49]

[x]: reference number of included study

Bold: the study received high risk of bias in the quality assessment

^a^ Anatomical site is relates to both hip and knee

**Table 5:** Clinical population studies. Prevalence and incidence rates of musculoskeletal extremity complaints in children by age group

	0–9 years of age		10–19 years of age	
	Incidence, %		Incidence, %	
Anatomic Region/Site	Per 1000 person years	Management rate per 100 encounters^a^	Per 1000 person years	Management rate per 100 encounters^a^
Upper extremity in general		1.30–1.38 [45]		1.38–4.55 [45]
Shoulder	0.0–0.7 [44]		1.4–4.8 [44]	
Arm	2.0–2.3 [44]		2.3–2.8 [44]	
Elbow	1.3–1.7 [44]		1.9–2.3 [44]	
Wrist/hand/finger	1.8–5.0 [44]		6.3–9.1 [44]	
Lower extremity in general		1.72–1.85 [45]		2.26–5.33 [45]
Hip/groin	4.0–4.9 [46]		2.1–2.2 [46]	
Thigh	4.1–4.2 [46]		4.3–5.1 [46]	
Knee	0.0–3.6 [46]		0.8–17.0 [46]	
Ankle/ft	1.9–9.2 [46]		5.0–16.2 [46]	

[x]: reference number of included study

^a^ Management rate per 100 encounters: diagnoses recorded at general practitioners per 100 consecutive encounters

**Table 6:** Results from three population based studies including a distinction between traumatic or non-traumatic mode of onset

Anatomic Region/Site	Non-traumatic	Traumatic	Ratio Non-traumatic: Traumatic
Upper extremity in general	0.04 [14]^a^ 0.2 [14]^b^	0.1 [14]^a^ 0.3 [14]^b^	1:2.5 1:1.5
Shoulder	0.03 [39]^c^	0.03 [39]^c^	1:1
Elbow	0.01 [39]^c^	0.02 [39]^c^	1:2
Hand/wrist/fingers	0.01 [39]^c^	0.13 [39]^c^	1:13
Lower extremity in general	12.3 [15]^b^ 0.7 [14]^a^ 3.2 [14]^b^	6.0 [15]^b^ 0.3 [14]^a^ 1.1 [14]^b^	1:0.5 1:0.4 1:0.3
Hip	2.7 [15]^b^ 0.05 [39]^c^	0.7 [15]^b^ 0.01 [39]^c^	1:0.3 1:0.2
Thigh	9.5 [15]^b^ 0.04 [39]^c^	0.9 [15]^b^ 0.03 [39]^c^	1:0.1 1:0.8
Knee	10.3 [15]^b^ 0.31 [39]^c^	2.1 [15]^b^ 0.09 [39]^c^	1:0.2 1:0.3
Shin	0.11 [39]^c^	0.01 [39]^c^	1:0.01
Ankle/Foot	8.8 [15]^b^ 0.46 [39]^c^	1.8 [15]^b^ 0.25 [39]^c^	1:0.2 1:0.5

[x]: reference number of included study

^a^ weekly incidence

^b^ weekly prevalence

^c^ Incidence Rate - per 1000 physical activity units

### General population studies

In both the younger (aged 0–12) and the older children (aged 10–19), lower extremity complaints were more common than upper extremity complaints, and ankle/ft and knee were the most frequent sites of MEC. Among the younger children, two studies reported ankle/ft complaints to be about twice as frequent as knee complaints [39, 54], whereas the last study reported almost similar prevalence rates for the two sites [15] (Table 3). Among the older children, five of the six included studies reported 0.2 to 2.8 times more knee complaints than ankle/ft complaints [40, 42, 43, 50, 52] (Table 4). In the upper extremities, wrist/hand/fingers was the most common site of complaint in younger children [39, 54] (Table 3), whereas shoulder complaints were more common among older children [42, 50, 52] (Table 4). The least frequent anatomical site of complaint reported in both younger and older children was the elbow (Tables 3 and 4).

### Clinical population studies

Also in the clinical population studies, lower extremity complaints were more frequent than complaints from the upper extremities [44–46]. Two of the three clinical population studies were based on data from the same cohort of children, but one reported on upper extremities [44] and the other on lower [46]. In the younger children the incidence rate of ankle/ft complaints was about three times higher than for the knee, whereas the incidence rates were almost equal for the two pain sites in the older children [46]. In the upper extremity, wrist/hand/fingers was the most common site in both age groups [44]. The least frequent site of complaint was the shoulder in the younger group, and shoulder and hip/groin in the older age group (Table 5).

### Traumatic versus non-traumatic complaints

In the younger children, three of the general population studies classified the complaints of the lower extremities into traumatic or non-traumatic mode of onset [14, 15, 39], but two of these were based on the same cohort of children [14, 39]. All three reported about two times more non-traumatic complaints compared to traumatic complaints (1:0.5 [15], 1:0.3 and 1:0.4 [14], respectively). One of the studies also reported mode of onset in upper extremities and found the opposite relationship, with non-traumatic complaints less frequently reported than traumatic complaints (1:1.5 and 1:2.5, for prevalence and incidence respectively) [14] (Table 6). There were no reports of mode of onset in other studies, neither in the older age group nor in the clinical population studies.

### General population studies versus clinical population studies

Comparing lower and upper extremities in general, MEC were reported up to ten times more often from the lower than from the upper extremities (mean ratio 1:4.0; range 1:1.1 to 1:10.3) in the general population studies (Tables 3 and 4), whereas the difference was much smaller in the clinical population studies with ratios from 1:1.2 to 1:1.6 (mean 1:1.4) (Table 5). In the general population studies, the difference was more significant in the younger children (mean ratio 1:5.1; range 1:2.3 to 1:10.3) than in the older children (mean ratio 1:2.5; range 1:1.1 to 1:4.6).

### Consequences of quality

No pattern of differences in reporting could be detected between studies of high and low risk of bias.

## Discussion

In the general population studies, ankle/ft and knee were the most frequently reported anatomical complaint sites. We found relatively more ankle/ft complaints in the younger children and more knee complaints in the older age group, and this pattern has to our knowledge not been reported before. The dominance of ankle/ft and knee complaints was similar to what was found in a review of sports-related injuries in children and adolescents [11] indicating either that many of these complaints often are related to sport, or that sports participation actually reveals otherwise unnoticed injuries. A possible explanation for the changing complaint pattern with age could be the development of the musculoskeletal system due to pubertal growth including a general rapid physical growth [56]. One consequence of this could be that the calcaneal growth plate is stressed by the Achilles tendon (Sever’s disease) in younger children whereas, when the child matures, the growth of the immature skeleton more commonly leads to apophysitis around the anterior knee located to the tibial tubercle or the inferior patellar pool, leading to more knee complaints in older child. Another shift with age was the relatively high occurrence of shoulder complaints in older children, whereas this was very rare in younger children. One explanation could be that teenagers in general spend more time in front of desktops or tablets, which might lead to posture related pain especially in the neck/shoulder region [57]. Whether these complaints actually relate to the shoulder joint or whether it is more related to the upper spine or the trapezius muscle is uncertain. It might be difficult for children and adolescents to distinguish between those two sources of pain, and it has been documented previously that neck and back pain increase with age during adolescence [4, 58]. If that is the case, the self-reported shoulder complaints are overestimated. To gain more reliable knowledge in this area, detailed questionnaires, including mannequin drawings, or interviews should be used in future studies.

In the clinical population studies, a similar pattern was seen for the lower extremities, but wrist/hand/fingers was the second most frequently reported site among young children, and the third most frequent in the older age group. However, this was based on only one study [44], albeit of high quality. In a review of fractures, Clark et al. also reported this area (the distal radius, fingers and carpal bones) to be the most common [16], indicating a certain susceptibility to both severe and more trivial injuries in the hand and wrist.

We found that lower extremity complaints were much more common than upper extremity complaints in the general population studies, whereas the clinical population studies showed a smaller difference in frequency between the two regions. Furthermore, the three studies reporting mode of onset, reported twice as many non-traumatic compared to traumatic complaints in the lower extremities. These two findings might indicate an overrepresentation of less severe complaints and/or more non-traumatic complaints in the lower extremities. In sports medicine, data collection traditionally has been based on diagnoses from emergency departments or among athletes requiring medical attention or time-loss definitions, and therefore reports of minor complaints have not been collected [59, 60]. In 1989, Backx et al. found that only 31% of all sports injuries led to health care usage [61], e.g. were serious enough to warrant consultation.

Recently Clarsen et al. developed a new tool to register overuse injuries in sport and compared it to the traditional time-loss registration of injuries. They found a completely different pattern of injuries in sport with especially more shoulder, knee and low back injuries [62], which support the thesis that some shoulder and knee complaints do not reach the threshold for health care consultation. In relation to reports of shoulder complaints, we also noted that prevalence rates were relatively high compared to incidence rates, which might indicate that these complaints are more long-lasting than other complaints which have smaller differences between prevalence and incidence rates. However, it could also be due to the misclassification mentioned earlier and actually relate to the neck or Trapezius area.

Most of the studies were conducted in the northern part of Europe and different patterns of complaints could possibly be present in other parts of the world. The type of injury might be related to the prevalent type of sport, and obviously there are large geographical differences in sport activities. Likewise, the threshold for reporting pain and the pattern of health care consumption are strongly culturally dependent, and therefore caution should be exercised when extrapolating results to other parts of the world.

The largest challenge of this review was the heterogeneity of outcomes; pooling of results to obtain reliable combined estimates of prevalence and incidence was impossible to conduct. The included 22 articles in this review used 14 different outcome measures, and this prevented meaningful combined estimates of prevalence or incidence.

The variation in outcome measurement and lack of knowledge in this area, also made it difficult to assess the risk of bias in the ‘Outcome measurement’ domain, which might have resulted in a too positive bias rating. Although the QUIPS tool has been validated [25] the modified version tool used in this review has not, which might be considered as a limitation of this review. Finally, it would have been appropriate to repeat the literature search in other databases. However, it is our experience that epidemiological articles can be found in the two databases that were used, MEDLINE and EMBASE, and therefore we do believe that the performed literature search can be justified, although we realize that potential relevant articles can have be missed.

Another issue in relation to heterogeneity is that there are challenges with use of questionnaires within this age group. In seven of the 19 general population studies, questionnaires or text messages were answered by parents [14, 39, 40, 47, 49, 51, 53]. Therefore, the concordance between parents’ and children’s reporting is important to understand. In general, agreement between children and parents is poor, especially in relation to minor complaints, whereas more severe complaints result in a better agreement [63–65]. Another potential problem is that the questionnaires have rarely been validated in the appropriate age groups, and finally the risk of recall bias might differ between children and adults. Harel et al. evaluated recall periods of 2 weeks to 12 months in children and adolescents in the US and found that reporting of severe injuries are not strongly affected by recall bias [66]. Another study by Moshiro et al., with a recall period of twelve months, found the same in an all-age cohort from Tanzania [67]. On the other hand, Harel et al. also concluded that reporting of minor complaints *are* affected by memory, especially if the recall period exceeds five months [66]. This indicates that to collect reliable estimates of minor symptoms the recall period needs to be relatively short. On this note, severity of complaint also needs to be considered, and this was often not the case in the included studies. In four of the 19 general population studies, participants were asked to categorize the complaints according to either severity or frequency, e.g. if the complaint was experienced never, once a month or once a week [15, 38, 41, 42], and in three other studies, consulting a physician was used as a measure of severity [47, 50, 54], but in the remaining studies no severity measures were reported.

Thus, in future studies data should be collected in a standardized way with due consideration to demarcation of the area, the parent/child reporting relationship, the severity and the frequency of pain. It became apparent during this review, that there is a strong need for better and more homogenous data collection methods. We therefore think it is time to make an effort to standardize future studies in relation to data collection with due to consideration to demarcation of the area, the severity and frequency of pain, and the parent/child reporting relationship. Furthermore, common age group definitions should be agreed upon. This could for example be obtained through a Delphi process followed by a cross-cultural adaption of age-standardized questionnaires.

Fortunately, the profound heterogeneity did not affect the comparisons between anatomical sites, age groups etc. However, another difficulty encountered was that different terms of MEC complicated the literature search and therefore we are aware that there might be articles missing. Hopefully, the assistance of a research librarian and a wide search strategy has minimized the effect of this difficulty.

## Conclusion

In general, ankle/ft and knee were the most frequent sites of musculoskeletal extremity complaints regardless of age and type of population. However, in the general population studies, there were relatively more non-traumatic complaints of the lower extremities than in the clinical population studies, indicating a large amount of non-traumatic low intensity complaints in the general population that do not reach threshold for consultation.

We intended to describe the prevalence and incidence of musculoskeletal extremity complaints in children and adolescents, but a meta-analysis, or even a simple overall description of prevalence and incidence, was not feasible, due to study heterogeneity, primarily related to outcome. Future research should use standardised and validated outcome measures and investigate the possible consequences of the low intensity complaints in large longitudinal cohorts to establish if there is a potential for prevention of long-term sequelae through early detection and intervention.

## Additional files

Additional file 1: description of the literature search strategy (PDF 51 kb)

Additional file 2: overview of the tool used for the quality assessment (PDF 72 kb)

Additional file 3: prima checklist (PDF 119 kb)

## Abbreviation

KBD: Kristina Boe Dissing
LH: Lise Hestbæk
MEC: Musculoskeletal extremity complaint
QUIPS: Quality In Prognosis Studies
SF: Signe Fuglkjær

## References

[1] Disease GBD, Injury I, Prevalence C (2016). Global, regional, and national incidence, prevalence, and years lived with disability for 310 diseases and injuries, 1990-2015: a systematic analysis for the global burden of disease study 2015. Lancet.

[2] Martin BI, Deyo RA, Mirza SK, Turner JA, Comstock BA, Hollingworth W, Sullivan SD (2008). Expenditures and health status among adults with back and neck problems. JAMA.

[3] Jeffries LJ, Milanese SF, Grimmer-Somers KA (2007). Epidemiology of adolescent spinal pain: a systematic overview of the research literature. Spine (Phila Pa 1976).

[4] Aartun E, Hartvigsen J, Wedderkopp N, Hestbaek L (2014). Spinal pain in adolescents: prevalence, incidence, and course: a school-based two-year prospective cohort study in 1,300 Danes aged 11-13. BMC Musculoskelet Disord.

[5] Dissing KB, Hestbaek L, Hartvigsen J, Williams C, Kamper S, Boyle E, Wedderkopp N (2017). Spinal pain in Danish school children - how often and how long? The CHAMPS study-DK. BMC Musculoskelet Disord.

[6] Janssen I, Leblanc AG (2010). Systematic review of the health benefits of physical activity and fitness in school-aged children and youth. Int J Behav Nutr Phys Act.

[7] Ekblom B, Astrand PO (2000). Role of physical activity on health in children and adolescents. Acta Paediatr.

[8] Andersen LB, Riddoch C, Kriemler S, Hills AP (2011). Physical activity and cardiovascular risk factors in children. Br J Sports Med.

[9] Expert Panel on Integrated Guidelines for Cardiovascular H, Risk Reduction in C, Adolescents, National Heart L, Blood I. Expert panel on integrated guidelines for cardiovascular health and risk reduction in children and adolescents: summary report, Pediatrics. 2011. 128 Suppl 5:S213–56. 10.1542/peds.2009-2107C PMC4536582 22084329

[10] Fuller CW, Ekstrand J, Junge A, Andersen TE, Bahr R, Dvorak J, Hagglund M, McCrory P, Meeuwisse WH (2006). Consensus statement on injury definitions and data collection procedures in studies of football (soccer) injuries. Clin J Sport Med.

[11] Caine D, Caine C, Maffulli N (2006). Incidence and distribution of pediatric sport-related injuries. Clinical journal of sport medicine : official journal of the Canadian Academy of Sport Medicine.

[12] Fernandez WG, Yard EE, Comstock RD (2007). Epidemiology of lower extremity injuries among U.S. high school athletes. Acad Emerg Med Off J Soc Acad Emerg Med.

[13] van Mechelen W, Hlobil H, Kemper HC (1992). Incidence, severity, aetiology and prevention of sports injuries. A review of concepts. Sports medicine (Auckland, NZ).

[14] Jespersen E, Holst R, Franz C, Rexen CT, Klakk H, Wedderkopp N (2014). Overuse and traumatic extremity injuries in schoolchildren surveyed with weekly text messages over 2.5 years. Scand J Med Sci Sports.

[15] El-Metwally A, Salminen JJ, Auvinen A, Kautiainen H, Mikkelsson M (2006). Risk factors for traumatic and non-traumatic lower limb pain among preadolescents: a population-based study of Finnish schoolchildren. BMC Musculoskelet Disord.

[16] Clark EM (2014). The epidemiology of fractures in otherwise healthy children. Current osteoporosis reports.

[17] Doherty C, Delahunt E, Caulfield B, Hertel J, Ryan J, Bleakley C (2014). The incidence and prevalence of ankle sprain injury: a systematic review and meta-analysis of prospective epidemiological studies. Sports medicine (Auckland, NZ).

[18] Seah R, Mani-Babu S (2011). Managing ankle sprains in primary care: what is best practice? A systematic review of the last 10 years of evidence. Br Med Bull.

[19] Kerkhoffs GM, Struijs PA, Marti RK, Assendelft WJ, Blankevoort L, van Dijk CN (2002). Different functional treatment strategies for acute lateral ankle ligament injuries in adults. The Cochrane database of systematic reviews.

[20] Gallo V, Egger M, McCormack V, Farmer PB, Ioannidis JP, Kirsch-Volders M, Matullo G, Phillips DH, Schoket B, Stromberg U (2011). STrengthening the reporting of OBservational studies in epidemiology-molecular epidemiology (STROBE-ME): an extension of the STROBE statement. Eur J Epidemiol.

[21] Dreyer NA, Velentgas P, Westrich K, Dubois R (2014). The GRACE checklist for rating the quality of observational studies of comparative effectiveness: a tale of hope and caution. J Manag Care Spec Pharm.

[22] Meader N, King K, Llewellyn A, Norman G, Brown J, Rodgers M, Moe-Byrne T, Higgins JP, Sowden A, Stewart G (2014). A checklist designed to aid consistency and reproducibility of GRADE assessments: development and pilot validation. Systematic reviews.

[23] [http://methods.cochrane.org/bias/sites/methods.cochrane.org.bias/files/uploads/Tool to Assess Risk of Bias in Cohort Studies.pdf ’data accessed’].

[24] Higgins JP, Altman DG, Gotzsche PC, Juni P, Moher D, Oxman AD, Savovic J, Schulz KF, Weeks L, Sterne JA (2011). The Cochrane Collaboration's tool for assessing risk of bias in randomised trials. BMJ.

[25] Hayden JA, van der Windt DA, Cartwright JL, Cote P, Bombardier C (2013). Assessing bias in studies of prognostic factors. Ann Intern Med.

[26] Sanderson S, Tatt ID, Higgins JP (2007). Tools for assessing quality and susceptibility to bias in observational studies in epidemiology: a systematic review and annotated bibliography. Int J Epidemiol.

[27] von Elm E, Altman DG, Egger M, Pocock SJ, Gotzsche PC, Vandenbroucke JP, Initiative S (2007). The Strengthening the reporting of observational studies in epidemiology (STROBE) statement: guidelines for reporting observational studies. Lancet.

[28] Picavet HS, Schouten JS (2003). Musculoskeletal pain in the Netherlands: prevalences, consequences and risk groups, the DMC(3)-study. Pain.

[29] Kaspiris A, Zafiropoulou C (2009). Growing pains in children: epidemiological analysis in a Mediterranean population. Joint, bone, spine : revue du rhumatisme.

[30] Sorensen L, Larsen SE, Rock ND (1996). The epidemiology of sports injuries in school-aged children. Scand J Med Sci Sports.

[31] Abou El-Soud AM, Gaballa HA, Ali MA (2012). Prevalence of osteochondritis among preparatory and primary school children in an Egyptian governorate. Rheumatol Int.

[32] El-Metwally A, Salminen JJ, Auvinen A, Kautiainen H, Mikkelsson M (2005). Lower limb pain in a preadolescent population: prognosis and risk factors for chronicity--a prospective 1- and 4-year follow-up study. Pediatrics.

[33] Menz HB, Jordan KP, Roddy E, Croft PR (2010). Characteristics of primary care consultations for musculoskeletal foot and ankle problems in the UK. Rheumatology.

[34] Siivola SM, Levoska S, Latvala K, Hoskio E, Vanharanta H, Keinanen-Kiukaanniemi S (2004). Predictive factors for neck and shoulder pain: a longitudinal study in young adults. Spine.

[35] Adams AL, Kessler JI, Deramerian K, Smith N, Black MH, Porter AH, Jacobsen SJ, Koebnick C (2013). Associations between childhood obesity and upper and lower extremity injuries. Inj Prev.

[36] Diepenmaat AC, van der Wal MF, de Vet HC, Hirasing RA (2006). Neck/shoulder, low back, and arm pain in relation to computer use, physical activity, stress, and depression among Dutch adolescents. Pediatrics.

[37] Ehrmann Feldman D, Shrier I, Rossignol M, Abenhaim L (2002). Risk factors for the development of neck and upper limb pain in adolescents. Spine (Phila Pa 1976).

[38] Hoftun GB, Romundstad PR, Zwart JA, Rygg M (2011). Chronic idiopathic pain in adolescence--high prevalence and disability: the young HUNT study 2008. Pain.

[39] Jespersen E, Rexen CT, Franz C, Moller NC, Froberg K, Wedderkopp N (2015). Musculoskeletal extremity injuries in a cohort of schoolchildren aged 6-12: a 2.5-year prospective study. Scand J Med Sci Sports.

[40] Krul M, van der Wouden JC, Schellevis FG, van Suijlekom-Smit LW, Koes BW (2009). Musculoskeletal problems in overweight and obese children. Ann Fam Med.

[41] Mikkelsson M, Salminen JJ, Kautiainen H (1997). Non-specific musculoskeletal pain in preadolescents. Prevalence and 1-year persistence. Pain.

[42] Rathleff MS, Roos EM, Olesen JL, Rasmussen S (2013). High prevalence of daily and multi-site pain--a cross-sectional population-based study among 3000 Danish adolescents. BMC Pediatr.

[43] Shrier I, Ehrmann-Feldman D, Rossignol M, Abenhaim L (2001). Risk factors for development of lower limb pain in adolescents. J Rheumatol.

[44] Bot SD, van der Waal JM, Terwee CB, van der Windt DA, Schellevis FG, Bouter LM, Dekker J (2005). Incidence and prevalence of complaints of the neck and upper extremity in general practice. Ann Rheum Dis.

[45] Henschke N, Harrison C, McKay D, Broderick C, Latimer J, Britt H, Maher CG (2014). Musculoskeletal conditions in children and adolescents managed in Australian primary care. BMC Musculoskelet Disord.

[46] van der Waal JM, Bot SD, Terwee CB, van der Windt DA, Schellevis FG, Bouter LM, Dekker J (2006). The incidences of and consultation rate for lower extremity complaints in general practice. Ann Rheum Dis.

[47] Hulsegge G, van Oostrom SH, Picavet HS, Twisk JW, Postma DS, Kerkhof M, Smit HA, Wijga AH (2011). Musculoskeletal complaints among 11-year-old children and associated factors: the PIAMA birth cohort study. Am J Epidemiol.

[48] Molgaard C, Rathleff MS, Simonsen O (2011). Patellofemoral pain syndrome and its association with hip, ankle, and foot function in 16- to 18-year-old high school students: a single-blind case-control study. J Am Podiatr Med Assoc.

[49] Abujam B, Mishra R, Aggarwal A (2014). Prevalence of musculoskeletal complaints and juvenile idiopathic arthritis in children from a developing country: a school-based study. Int J Rheum Dis.

[50] Auvinen JP, Paananen MV, Tammelin TH, Taimela SP, Mutanen PO, Zitting PJ, Karppinen JI (2009). Musculoskeletal pain combinations in adolescents. Spine (Phila Pa 1976).

[51] Bishop JL, Northstone K, Emmett PM, Golding J (2012). Parental accounts of the prevalence, causes and treatments of limb pain in children aged 5 to 13 years: a longitudinal cohort study. Arch Dis Child.

[52] Smedbraten BK, Natvig B, Rutle O, Bruusgaard D (1998). Self-reported bodily pain in schoolchildren. Scand J Rheumatol.

[53] Słowińska IKM, Jednacz E, Mańczak M, Rutkowska-Sak LRF (2015). Pain associated with the musculoskeletal system in children from Warsaw schools. Reumatologia.

[54] Verhagen E, Collard D, Paw MC, van Mechelen W (2009). A prospective cohort study on physical activity and sports-related injuries in 10-12-year-old children. Br J Sports Med.

[55] Feldman DE, Shrier I, Rossignol M, Abenhaim L (2001). Risk factors for the development of low back pain in adolescence. Am J Epidemiol.

[56] Patton GC, Viner R (2007). Pubertal transitions in health. Lancet.

[57] Brink Y, Louw QA (2013). A systematic review of the relationship between sitting and upper quadrant musculoskeletal pain in children and adolescents. Man Ther.

[58] Kjaer P, Wedderkopp N, Korsholm L, Leboeuf-Yde C (2011). Prevalence and tracking of back pain from childhood to adolescence. BMC Musculoskelet Disord.

[59] Fuller CW, Ekstrand J, Junge A, Andersen TE, Bahr R, Dvorak J, Hagglund M, McCrory P, Meeuwisse WH (2006). Consensus statement on injury definitions and data collection procedures in studies of football (soccer) injuries. Br J Sports Med.

[60] Bahr R (2009). No injuries, but plenty of pain? On the methodology for recording overuse symptoms in sports. Br J Sports Med.

[61] Backx FJ, Erich WB, Kemper AB, Verbeek AL (1989). Sports injuries in school-aged children. An epidemiologic study. Am J Sports Med.

[62] Clarsen B, Myklebust G, Bahr R (2013). Development and validation of a new method for the registration of overuse injuries in sports injury epidemiology: the Oslo sports trauma research Centre (OSTRC) overuse injury questionnaire. Br J Sports Med.

[63] Sundblad GM, Saartok T, Engstrom LM (2006). Child-parent agreement on reports of disease, injury and pain. BMC Public Health.

[64] Singer AJ, Gulla J, Thode HC (2002). Parents and practitioners are poor judges of young children's pain severity. Acad Emerg Med Off J Soc Acad Emerg Med.

[65] Kroner-Herwig B, Morris L, Heinrich M, Gassmann J, Vath N (2009). Agreement of parents and children on characteristics of pediatric headache, other pains, somatic symptoms, and depressive symptoms in an epidemiologic study. Clin J Pain.

[66] Harel Y, Overpeck MD, Jones DH, Scheidt PC, Bijur PE, Trumble AC, Anderson J (1994). The effects of recall on estimating annual nonfatal injury rates for children and adolescents. Am J Public Health.

[67] Moshiro C, Heuch I, Astrom AN, Setel P, Kvale G (2005). Effect of recall on estimation of non-fatal injury rates: a community based study in Tanzania. Inj Prev.

[68] Slowinska I, Kwiatkowska M, Jednacz E, Manczak M, Rutkowska-Sak L, Raciborski F (2015). Pain associated with the musculoskeletal system in children from Warsaw schools. Reumatologia.

