# Supplementary material for: Prevalence and incidence of musculoskeletal extremity complaints in children and adolescents. A systematic review
Source: BMC Musculoskelet Disord. 2017 Oct 18;18:418. doi: 10.1186/s12891-017-1771-2 (PMC5648427; doi:10.1186/s12891-017-1771-2)
Supplement: Supplementary file 1 — description of the literature search strategy (PDF 51 kb) [file 12891_2017_1771_MOESM1_ESM.pdf]

Supplementary file 1

The literature search were performed in Pubmed and Embase

Search in pudmed

(("musculoskeletal diseases"[MeSH Terms] OR ("musculoskeletal"[All Fields] AND "diseases"[All Fields]) OR "musculoskeletal diseases"[All Fields] OR ("musculoskeletal"[All Fields] AND "disorder"[All Fields]) OR "musculoskeletal disorder"[All Fields]) OR (musculoskeletal[All Fields] AND ("wounds and injuries"[MeSH Terms] OR ("wounds"[All Fields] AND "injuries"[All Fields]) OR "wounds and injuries"[All Fields] OR "injury"[All Fields])) OR ("musculoskeletal pain"[MeSH Terms] OR ("musculoskeletal"[All Fields] AND "pain"[All Fields]) OR "musculoskeletal pain"[All Fields]) OR (musculoskeletal[All Fields] AND complaint[All Fields])) AND (("epidemiology"[Subheading] OR "epidemiology"[All Fields] OR "prevalence"[All Fields] OR "prevalence"[MeSH Terms]) OR ("epidemiology"[Subheading] OR "epidemiology"[All Fields] OR "incidence"[All Fields] OR "incidence"[MeSH Terms])) AND (("extremities"[MeSH Terms] OR "extremities"[All Fields]) OR ("extremities"[MeSH Terms] OR "extremities"[All Fields] OR "extremity"[All Fields])) AND (("child"[MeSH Terms] OR "child"[All Fields] OR "children"[All Fields]) OR ("adolescent"[MeSH Terms] OR "adolescent"[All Fields] OR "adolescence"[All Fields]) OR ("adolescent"[MeSH Terms] OR "adolescent"[All Fields] OR "adolescents"[All Fields]) OR toddler[All Fields] OR ("adolescent"[MeSH Terms] OR "adolescent"[All Fields] OR "teenager"[All Fields]) OR ("pediatrics"[MeSH Terms] OR "pediatrics"[All Fields] OR "paediatric"[All Fields]))

Search in Embase

Musculoskeletal disease [subject heading]	Limb [subject heading]	Child [subject heading]
OR	OR	OR
Musculoskeletal [abstract]	Limb [abstract]	Adolescents [subject heading]
OR	OR	OR
Musculoskeletal injury [subject heading]	Extremit* [abstract]	Child [abstract]
