# Supplementary material for: Prevalence and incidence of musculoskeletal extremity complaints in children and adolescents. A systematic review
Source: BMC Musculoskelet Disord. 2017 Oct 18;18:418. doi: 10.1186/s12891-017-1771-2 (PMC5648427; doi:10.1186/s12891-017-1771-2)
Supplement: Supplementary file 2 — overview of the tool used for the quality assessment (PDF 72 kb) [file 12891_2017_1771_MOESM2_ESM.pdf]

Author:
Year:
Rater:

Domain	Description
1. Study participation<br>a. Adequate participation in the study by eligible persons<br>b. Description of the source population or population of interest<br>c. Description of the baseline study sample<br>d. Adequately description of the sampling frame and recruitment.<br>e. Adequately description of the period and place of recruitment<br>f. Adequate description of inclusion and exclusion criteria	a. Yes/no<br>b. Yes/no<br>c. Yes/no<br>d. Yes/no<br>e. Yes/no<br>f. Yes/no
2. Study Attrition<br>A. Adequate respons rate for study participation<br>B. Description of attempts to collect information on participants of people who dropped out.<br>C. Reasons for loss of follow up are provided.<br>D. Adequate description of participants lost to follow-up.<br>E. There are no important differences between participants who completed the study and those you did not.	a. Yes/no<br>b. Yes/no<br>c. Yes/no<br>d. Yes/no<br>e. Ok?
3. Outcome measurement<br>a. A clear definition of outcome is provided.<br>b. Method of outcome measurement used is adequately valid and reliable<br>c. Method and setting of outcome measurements is the same for all study participants	a. Yes/no<br>b. Yes/no<br>c. Yes/no

4. Analysis and rapporting<br>a. To Access prevalence rates /. Sufficient presentation of data.<br>b. There is no selective reporting of results	a. Yes/no<br>b. Ok?

Potentiel bias

	**Judgement**
**1. Study participation**<br>The study sample adequately represents the population of interest	Yes<br>Partly<br>No<br>Not reported/unsure
**2. Study Attrition**<br>The study data available (i.e. participants not lost to follow-up) adequately represent the study sample	**Judgement**<br>Yes<br>Partly<br>No<br>Not reported/unsure
**3. Outcome measurement**<br>The outcome of interest is measured in a similar way for all participants.	**Judgement**<br>Yes<br>Partly<br>No<br>Not reported/unsure
**4. Analysis and rapporting**<br>The statistical analysis is appropriate,<br>and all primary outcomes are reported	**Judgement**<br>Yes<br>Partly<br>No<br>Not reported/unsure

Additional comments:
